# Health effects of carbonaceous PM2.5 compounds from residential fuel combustion and road transport in Europe

**DOI:** 10.1038/s41598-024-51916-9

**Published:** 2024-01-17

**Authors:** Niki Paisi, Jonilda Kushta, Andrea Pozzer, Angelos Violaris, Jos Lelieveld

**Affiliations:** 1 Climate and Atmosphere Research Center (CARE-C), The Cyprus Institute, 2121 Nicosia, Cyprus; 2 Present Address: Department of Atmospheric Chemistry, Max Planck Institute for Chemistry, 55128 Mainz, Germany

**Keywords:** Environmental impact, Atmospheric chemistry

## Abstract

Exposure to fine particulate matter (PM2.5) is associated with an increased risk of morbidity and mortality. In Europe, residential fuel combustion and road transport emissions contribute significantly to PM2.5. Toxicological studies indicate that PM2.5 from these sources is relatively more hazardous, owing to its high content of black and organic carbon. Here, we study the contribution of the emissions from these sectors to long-term exposure and excess mortality in Europe. We quantified the impact of anthropogenic carbonaceous aerosols on excess mortality and performed a sensitivity analysis assuming that they are twice as toxic as inorganic particles. We find that total PM2.5 from residential combustion leads to 72,000 (95% confidence interval: 48,000–99,000) excess deaths per year, with about 40% attributed to carbonaceous aerosols. Similarly, road transport leads to about 35,000 (CI 23,000–47,000) excess deaths per year, with 6000 (CI 4000–9000) due to carbonaceous particles. Assuming that carbonaceous aerosols are twice as toxic as other PM2.5 components, they contribute 80% and 37%, respectively, to residential fuel combustion and road transport-related deaths. We uncover robust national variations in the contribution of each sector to excess mortality and emphasize the importance of country-specific emission reduction policies based on national characteristics and sectoral shares.

## Introduction

Air pollution contributes most to environmental health hazards and mortality in Europe, and is responsible for thousands of excess deaths per year from cardiovascular, respiratory and other non-communicable diseases^1^. Exposure to fine particulate matter (PM2.5), especially in urban areas, often reaches levels that surpass the recommended World Health Organization (WHO) safety thresholds^2^. Hazard ratio models developed from epidemiological cohort studies of long-term exposure to PM2.5 can describe the relationship between PM2.5 exposure and the risk of developing a disease leading to premature death. Such models include: the global exposure mortality model—GEMM^3,4^, and the meta-regression-Bayesian, regularized, trimmed—MR-BRT^5^. MR-BRT exposure response functions include cause-specific risk expressions for several diseases, such as ischemic heart disease, stroke (ischemic and hemorrhagic), chronic obstructive pulmonary disease, lung cancer and Type II diabetes among adults (population with age > 25 years), and acute lower respiratory tract infection among children (population under the age of 5)^6^. The relative risks differ between models, exposure ranges, age groups, and disease categories^7^. This depends on the exposure levels in the cohort studies, the statistical methods, and the causes of deaths included. For example, the GEMM addresses relative risks from non-communicable diseases and lower respiratory infections (NCD-LRI), encompassing the six causes of death considered in the MR-BRT functions. The six causes of death represent only part of the total mortality due to NCD-LRI, so that the estimates from the GEMM are higher than of the MR-BRT functions^7^. Another reason is that mortality at very high PM2.5 levels is estimated by using smoking and indoor air pollution studies in the MR-BRT functions whereas the GEMM only applies studies of outdoor air pollution^7^. Despite advances in understanding the mechanisms and relationship between air pollution and health impacts, the precision of excess mortality estimates will need to be further improved by additional epidemiological studies, especially in high-pollution and low-income settings^8^.

Epidemiological cohort studies increasingly indicate that carbonaceous particles are particularly hazardous to human health^9^. There is sufficient evidence from cohort studies of associations of all-cause and cardiopulmonary mortality with long-term average black carbon exposure^10^. Toxicological studies have also attempted to quantify the levels of harmfulness of different types of aerosols and have provided strong evidence that the different sub-components of PM2.5 are not equally noxious. Despite these indications, the current risk models do not account for the differential toxicity of PM2.5 sub-components, which is a fundamental limitation. It is also worth noting that toxicity studies do not quantify the accumulated long-term health effects of PM2.5 exposure.

A growing body of literature on PM2.5 has shown that anthropogenic pollutants and especially combustion-derived PM2.5 have a high potential to induce oxidative stress^11,12^. Oxidative stress occurs when there is an imbalance between reactive oxygen species (ROS) levels and the antioxidant defenses of an organism^13^. ROS are oxygen-containing reactive species (e.g. superoxide (O2▪), hydrogen peroxide (H_2_O_2_), and hydroxyl radicals (OH▪)), that occur at low levels in living cells. They are part of the normal intracellular functioning and physiological redox signaling but can be damaging when in excess^14^. The oxidative potential of PM2.5 has been found to vary by a factor of eight depending on the sources of particles^15^. A long-term in situ measurement study in the central Mediterranean estimated that combustion sources, such as traffic and biomass burning, contributed 50–51% of the total oxidative potential in PM2.5, whereas those from natural sources and soil dust contributed about 13–14%^16^. This indicates that PM2.5 composition (depending on the emission source) is an essential characteristic that influences the health effects. Carbonaceous aerosols (e.g., black carbon (BC) and anthropogenic organics), which are common combustion-derived particles, have been consistently reported to have an increased ability to generate ROS, and can be assumed to be more hazardous to human health compared to inorganic components^12^. For example, Chirizzi et al.^17^ estimated a higher oxidative potential in PM2.5 that originated from road traffic and biomass combustion sources compared to Saharan dust particles, and attributed this outcome to the high carbon content at the specific site. Guascito et al.^18^ found that both cellular and intracellular oxidative stress indicators are strongly influenced by combustion sources. Grange et al.^19^ clearly identified that road traffic and carbonaceous wood combustion sources had the greatest mass-normalized oxidative potential, and was clearly the most potent source of PM2.5 in Switzerland. Daellenbach et al.^12^ found a three-fold higher oxidative potential in secondary organic aerosols from residential biomass burning and vehicular emissions in urban particles compared to rural ones. Jansen et al.^20^ reported that the health effects of a 1 μg/m^3^ increase in exposure are greater for BC particles than for generic PM2.5. Stafoggia et al.^21^ found a consistent association between BC exposure and mortality through analysis of several large European cohort studies. Similarly, Hoek et al.^22^ found a significant association between all-cause mortality and BC and NO_2_, which are both markers of combustion. Further, BC is usually co-emitted with organic compounds such as polycyclic aromatic hydrocarbons (PAHs), quinones, and water-soluble transition metals (e.g., Fe, Cu, and Mn). These highly redox-active species, especially from diesel exhausts and biomass combustion sources, have attracted attention due to their pronounced ability to generate ROS in target cells and their propensity to initiate a cascade of cell-damaging mechanisms that can potentially lead to disease^23^. However, the exact extent of their toxicity and their relationship with specific health endpoints have not yet been fully elucidated^23^.

In Europe, the largest emitters of fine particles are residential combustion for heating and cooking purposes and transportation (e.g. from diesel and gasoline exhausts), which are also common sources of organic particles and BC^24–26^. With growing urbanization and population ageing, human exposure to hazardous air pollutants is unlikely to decline, and excess mortality can be expected to increase^27,28^. It is, therefore, important to frequently measure the contribution of these “high toxicity” emission sectors to excess mortality, which will facilitate the design of future strategies in a more efficient way and assessment of the efficacy of current ones. Precise estimation of the hazards associated with PM2.5 exposure is fundamental for addressing this environmental health risk and for better communicating the impacts of air pollution to policy makers and the public.

In this study, we simulated atmospheric PM2.5 concentrations in Europe for a well-documented representative year (2015) to calculate the excess mortality from long-term PM2.5 exposure. Our main objective was to estimate the contribution of residential combustion and road transport sectors to excess mortality due to total PM2.5 exposure, by removing the respective emissions by 100%. This methodology, generally based on air quality models, can provide valuable insights for policy-making^29^. Secondly, we aimed to quantify the excess mortality attributable to anthropogenic secondary organic, primarily organic, and BC aerosols (here referred to as anthropogenic carbonaceous aerosols—aCA), and further, assess how much their contribution differs when these are assumed to be more detrimental to human health than other fine particles. Finally, we quantify the benefits that can be achieved in terms of avoidable excess mortality by reducing the emissions of the two sectors by 20% and under the two toxicity assumptions.

## Methodology

### Model set-up and input data

We simulated PM2.5 and its carbonaceous constituents for the year 2015 using the WRF-CHEM-v.3.9.1 air quality model^30^ European domain at a spatial resolution of 20 × 20km. The model configuration is based on previous studies, where modeling improvements specifically for organic aerosols were achieved^24,31–33^. The initial meteorological and boundary conditions were provided by the National Centers for Environmental Prediction Final Operational Global Analysis (NCEP-FNL) data, while the chemical boundary conditions were obtained from the global Model for Ozone And Related chemical Tracers—MOZART-4 at 1.9 × 1.9° resolution^34^. We use meteorological grid analysis nudging towards the NCEP-FNL meteorological fields. The mineral dust and biogenic emissions are calculated online, the latter being computed using the Model of Emissions of Gases and Aerosols from Nature version 2.1 (MEGAN2.1). The gas–phase chemistry was simulated with the Regional Atmospheric Chemical Model coupled with Modal Aerosol Dynamics for Europe (MADE) with the Volatility Basis Set (VBS) (RACM/MADE-VBS). The VBS is used to calculate secondary organic aerosol (SOA) formation, taking into account the semi-volatile nature of organic aerosols and ageing processes (homogeneous oxidation of organic carbon vapors by OH radicals) and significantly improves the model’s ability to reproduce observed OA concentrations^35,36^. The anthropogenic emissions were obtained from the EDGARv.5 global emission inventory (http://data.europa.eu/89h/377801af-b094-4943-8fdc-f79a7c0c2d19)^37^, available at 0.1 × 0.1° resolution. Those were aggregated into sectoral emissions for the transportation (including railways, pipelines and road transportation) and residential combustion sectors. Additional information regarding model performance is included in the Supplementary material.

### Excess mortality calculations

Excess mortality is defined as the steady‐state difference in the number of deaths between exposed and unexposed populations and refers to the number of deaths that would not have occurred without exposure over a defined period of time^7,8^. Excess mortality estimation requires a function or model that relates the exposure to ambient air pollution to the relative risk (RR)^8^. In this study, cause- and age-specific RRs from exposure to ambient PM2.5 pollution were calculated using the recently updated exposure–response function from the Global Burden of Disease Study, the meta-regression-Bayesian, regularized, trimmed (MR-BRT) tool^5^. The MR-BRT includes studies of household air pollution, secondhand smoking, as well as multiple studies performed at high ambient exposure settings^8^. It includes RRs for ischemic heart disease (IHD), stroke, chronic obstructive pulmonary disease (COPD), lower respiratory infections (LRI), lung cancer (LC), and type II diabetes (T2D) among adults (over 25 years old) and acute lower respiratory infections among children (below 5 years old). More detailed information regarding MR-BRT risk function can be found in a recent Global Burden of Disease report (GBD, 2021)^38^. Here, we only account for adult mortality to allow comparison with our results using the GEMM risk model (Supplementary material). RRs for IHD and stroke are age-dependent and sub-divided into 5-year intervals from 25 to over 80 years old.

The MR-BRT splines were scaled to the theoretical minimum risk exposure level (TMREL) of 4.15 μg/m^3^, the concentration below which we assume no excess risk. This is the average value of the TMREL uniform distribution, which ranges between 2.4 and 5.9 μg/m^3^ ^39^ and is the same as used in the GEMM. Then, the attributable fraction (AF) was calculated, which expresses the fraction of mortality that is attributed to air pollution and is expressed as (RR-1/RR). AF at each grid was combined with baseline mortality rates (BMR) and population data (POP) per disease j and age k to calculate adult (> 25 years old) excess mortality attributable to PM2.5 long-term exposure (Eq. 1). The calculations were performed at a 5 × 5km resolution over the European domain, based on the PM2.5 concentration obtained from our WRF-Chem simulations and then aggregated to the country level. The countries included in this study are summarized in Table 1. As a sensitivity analysis, we also estimated excess mortality using RRs from the Global Exposure Mortality Model (GEMM)^4^, which, unlike MR-BRT, was based on studies of only outdoor air pollution exposure, leading to higher relative risks than MR-BRT. More details about the methodology with GEMM are included in the Supplementary material. The results described here are based on MR-BRT unless otherwise stated.

**Table 1:** Population-weighted annual mean concentrations (in μg/m^3^) for PM2.5 and anthropogenic carbonaceous aerosols per country (BASE scenario). The top five highest levels are highlighted in bold.

Country	Pop.-weighted-mean PM2.5	Pop.-weighted-mean aCA	Country	Pop.-weighted-mean PM2.5	Pop.-weighted-mean aCA
Albania	14.5	1.9	Lithuania	11.9	2.1
Austria	14.2	2.3	Luxembourg	15.8	2.6
Belgium	15.6	2.6	Malta	12.5	1.3
Bosnia and Herzegovina	**18.4**	3.5	Moldova	11.0	2.0
Bulgaria	13.6	2.1	Montenegro	13.4	1.7
Belarus	10.1	1.5	Netherlands	13.0	1.8
Croatia	17.9	3.6	Norway	6.2	0.6
Cyprus	13.8	1.3	Poland	17.5	**3.9**
Czech Republic	**19.3**	**4.2**	Portugal	9.5	1.3
Denmark	10.8	1.5	Romania	14.6	2.8
Estonia	9.6	1.5	Serbia	**20.8**	**3.8**
Finland	8.2	1.3	Slovakia	15.5	2.5
France	12.1	2.0	Slovenia	17.4	3.1
Germany	13.9	2.1	Spain	9.3	1.4
Greece	14.1	1.8	Sweden	8.0	1.0
Hungary	**17.1**	**3.7**	Switzerland	14.1	2.2
Iceland	3.2	0.1	Turkey	**18.6**	3.3
Ireland	7.1	0.8	Ukraine	10.4	1.6
Italy	16.5	2.9	North Macedonia	14.9	2.2
Latvia	11.1	1.9	United Kingdom	10.6	1.6
Liechtenstein	**18.0**	**3.8**			

Population data were obtained from NASA’s Socioeconomic Data and Application Center (SEDAC), GPW-v4 (https://sedac.ciesin.columbia.edu). These were combined with country-level age information from the Global Burden of Disease^40^ to obtain age-specific gridded population estimates for the following age classes: above 25 years and at 5 year intervals: 25–29, 30–34, 35–39, 40–44, 45–49, 50–54, 55–59, 60–64, 65–69, 70–74, 75–79, and 80 plus. Age-specific baseline mortality rates for each disease were obtained from the Global Burden of Disease Results Tool (http://ghdx.healthdata.org/gbd-results-tool). Finally, we estimated the contribution of anthropogenic carbonaceous aerosols by multiplying the excess mortality due to total PM2.5 by the mass concentration ratio of anthropogenic carbonaceous aerosols to PM2.5 (Eq. (2)). The anthropogenic carbonaceous aerosols include BC, primary organic carbon and anthropogenic secondary organic carbon aerosols.

1 $$M_{PM2.5} \left( {x,y} \right) = \mathop \sum \limits_{j,k}^{ } \left( {BMR\left( {x,y} \right) \times POP\left( {x,y} \right) \times Af(j,k } \right)$$

2 $$M_{aCA} \left( {x,y} \right) = M_{PM2,5} \left( {x,y} \right) \times \left( {\frac{{aCA\left( {x,y} \right)}}{{PM2.5\left( {x,y} \right)}}} \right)$$

where M_PM2.5_ is the excess mortality due to PM2.5 exposure and M_aCA_ is the excess mortality due to anthropogenic carbonaceous aerosols. x and y describe the grid coordinates and j and k represent each disease category (i.e., IHD, STROKE, COPD, LC, LRI, and type II diabetes) and age group (i.e., 25–29, 30–34, 35–39, 40–44, 45–49, 50–54, 55–59, 60–64, 65–69, 70–74, 75–79, and 80 plus), respectively.

### Sectoral contribution scenarios and differential toxicity

Based on EDGARv5^41^, we selected two of the predominant PM2.5 emission sectors in Europe, residential combustion and road transport, which have been also identified as relatively high-toxicity PM2.5 sources^12^, and assessed their impact on excess mortality. In particular, we quantified their contribution with five sensitivity runs listed below. Although other sectors are also significant contributors to PM2.5 emissions (e.g., industry), due to limited time and computational resources, we restricted our simulations to the following:

- BASE: baseline simulation where all emissions are included in the model.
- RES100: scenario where all emissions from residential combustion are completely removed.
- TRA100: scenario where all emissions from road transport are completely removed.
- RES20: scenario where 20% of emissions from residential combustion are removed.
- TRA20: scenario where 20% of emissions from the road transport sector are removed.

The pollutants that are removed in each of the sensitivity runs include Carbon Monoxide (CO), Nitrogen Oxides (NOx), Sulfur Dioxide (SO_2_), Ammonia (NH_3_), Black Carbon (BC), Organic Carbon (OC), particulate matter (PM2.5 and PM10), and non-methane volatile organic compounds (NMVOC). The contribution of each sector to excess mortality was quantified by subtracting the excess mortality that resulted from RES100 and TRA100 scenarios from the one obtained from the BASE scenario. As this is an upper limit setting, and since the state of the atmosphere is dynamic and possibly not linearly affected by such emission reductions, we also estimated the excess mortality under a scenario in which emissions coming from residential (RES20) or transport (TRA20) sectors are reduced by 20%.

We then performed sensitivity analyses to account for the differential toxicity of anthropogenic carbonaceous aerosols in our excess mortality calculations. We estimate that the toxicity increases with inhaled dose/exposure^6^ and repeated the calculations (Eqs. 1–2) assuming that anthropogenic carbonaceous fine particles are twice as toxic as other inorganic PM2.5 and increased their fractional contribution two-fold, while we considered the total PM2.5 toxicity constant. This method was applied in all emission reduction scenarios to capture the potentially increased contribution of those carbonaceous aerosols to the sectoral share. While the toxicity assumption applied in this work was chosen based on the available in-vitro toxicity data, it does not provide an absolute scale of their increased harmfulness, but rather emphasizes their significant contribution to the health impacts of PM2.5.

## Results

### Exposure to ambient PM2.5

**Figure 1:**
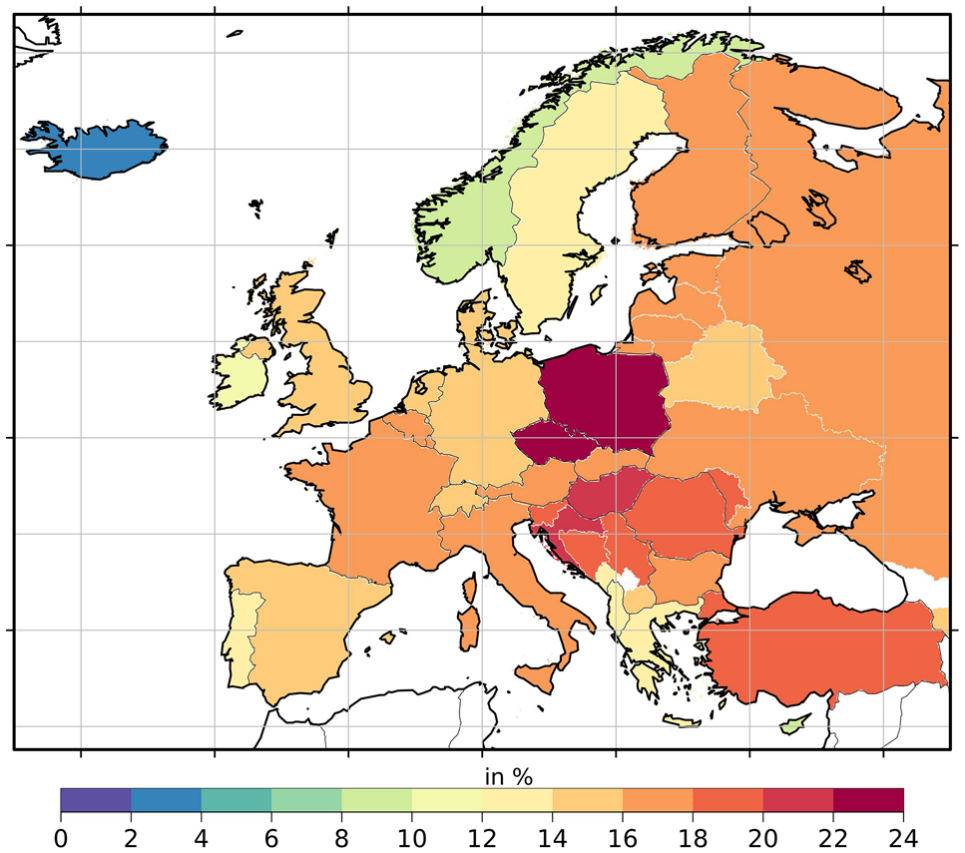
Fractional contribution of anthropogenic carbonaceous aerosols (aCA) to PM2.5 mass concentration (in % and population-weighted). This figure was produced with R software, version 4.3.2 (https://cran.r-project.org/bin/windows/base/).

Based on our baseline simulation, the contribution of anthropogenic carbonaceous aerosols to total PM2.5 concentrations ranges between 3.8 and 22.7% in EU countries (Fig. 1). In Central and Eastern Europe, including Hungary, Poland and the Czech Republic, carbon-rich PM2.5 is typically more abundant (near 23% of total PM2.5) compared to other countries, especially Northern European countries such as Norway and Iceland (3–10%) (Fig. 1). The population-weighted concentrations per country and per scenario, for both PM2.5 and anthropogenic carbonaceous aerosols, are included in Table S1. The per-country average population-weighted PM2.5 concentrations range between 3.2 and 20.8 μg/m^3^ over the wider European region. The populations of Serbia and the Czech Republic are found to be exposed to the highest annual-average PM2.5 concentrations (20.8 μg/m^3^ and 19.3 μg/m^3^, respectively), whereas the lowest exposure is found in Iceland (3.2 μg/m^3^). The population of Serbia has the highest exposure to anthropogenic carbonaceous aerosols, 4.2 μg/m^3^, which accounts for 17.2% of the total PM2.5 mass concentration. Northern European countries such as Sweden, Ireland, Norway, and Iceland have the lowest population-weighted concentrations of anthropogenic carbonaceous aerosols (1, 0.8, 0.6, 0.1 μg/m^3^, respectively).

### Excess mortality estimates

#### Baseline scenario

Overall, we find 281,000 (CI 207,000–317,000) excess deaths per year to be due to ambient PM2.5 exposure in the European region, of which 49,000 (CI 36,000–56,000) can be attributed to anthropogenic carbonaceous aerosols (assuming equal toxicity) (Table 2). This fraction of excess mortality increases to 98,000 (CI 72,000–112,000) annual excess deaths when we assume anthropogenic carbonaceous aerosols to be twice as toxic as other fine particles. The contributions of PM2.5 and anthropogenic carbonaceous aerosols to excess mortality for each disease category, assuming equal and two-fold toxicity are summarized in Table 2 (first column). The most important cause of death from exposure to ambient PM2.5 is cardiovascular diseases (IHD + Stroke), which cause 182,000 (CI 135,000–206,000) excess deaths per year. Respiratory diseases (COPD + LRI) and type II diabetes make similar contributions to total excess mortality from ambient PM2.5, with 34,000 (CI 25,000–41,000) and 38,000 (CI 26,000–39,000) excess deaths per year, respectively. We further estimate that around 27,000 (CI 21,000–30,000) annual excess deaths are due to lung cancer. We find that the contribution of anthropogenic carbonaceous aerosols to excess mortality varies markedly between countries and ranges between about 6% and 45% of the total, depending on toxicity assumption (Supplementary material, Table S2). Moreover, the annual excess mortality per disease category is quantified for each emission scenario for the whole domain (Table 2, columns 3–6) and are further discussed next.

**Table 2:** Annual adult excess mortality in thousand deaths (mean, 95% confidence interval) due to PM2.5 and anthropogenic carbonaceous aerosols per scenario, disease, and toxicity assumption.

Disease	BASE		TRA100	RES100	TRA20		RES20
PM2.5							
IHD	123 (88–141)		108 (79–121)	90 (67–97)	120 (86–138)		117 (84–133)
STROKE	59 (47–65)		52 (42–56)	43 (35–45)	58 (46–64)		56 (45–62)
COPD	23 (18–26)		20 (16–21)	16.7 (14–17)	22 (17–25)		22 (17–24)
T2D	38 (26–39)		29 (20–28)	29.7 (21–28)	37 (26–38)		36 (25–37)
LC	27 (21–30)		23 (19–25)	19.6 (16–20)	26 (21–29)		25 (20–28)
LRI	11 (7–15)		9 (6–12)	8 (5–10)	11 (7–15)		11 (7–14)
Anthropogenic carbonaceous aerosols (equal toxicity)							
IHD	22 (15–25)		19 (14–22)	10 (8–11)	21 (15–24)		19.4 (13.9–23)
STROKE	10 (8–2)		9 (7–10)	4.9 (4–5)	10 (8–11)		9.4 (7.4–10)
COPD	3.9 (3–4)		3.3 (2.6–3.7)	1.8 (1.5–1.9)	3.8 (3–4.3)		3.5 (2.7–4)
T2D	6.5 (4–7)		4.9 (3.4–5)	3.3 (2–3.2)	6 (4–7)		5.9 (4.1–6.1)
LC	4.7 (4–5)		4.1 (3.3–4.6)	2.2 (1.8–2.3)	4.6 (3.6–5.2)		4.2 (3.3–4.8)
LRI	2 (1–3)		1.6 (1.1–2.1)	0.9 (0.6–1.1)	2 (1–3)		1.7 (1.1–2.3)
Anthropogenic carbonaceous aerosols (two-fold toxicity)							
IHD	43 (31–50)		38 (28–43)	21 (15–22)	49 (30–49)		39 (28–45)
STROKE	21 (16–23)		18 (15–20)	13 (9–16)	20 (16–23)		19 (15–21)
COPD	8 (6–9)		6.7 (5.2–7.3)	3.7 (3–3.9)	8 (6–9)		7 (5–8)
T2D	13 (9–14)		9.8 (7–10)	6 (4.6–6.3)	12.8 (8.8–13.2)		12 (7–12.2)
LC	9 (7–11)		8.2 (6.5–9.1)	4.4 (3.6–4.6)	5 (7–10)		4 (7–10)
LRI	4 (2–5)		3.2 (2.3–4.3)	1.8 (1.2–2.3)	4 (2–5)		3 (2–5)

#### Residential combustion

**Figure 2:**
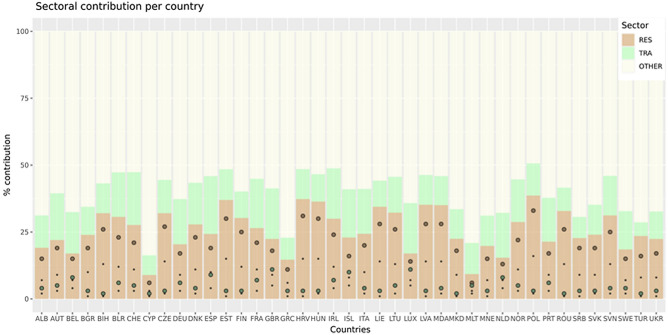
The contribution (in % of total) of residential (RES), road transport (TRA) and other (OTHER) sectors to excess mortality due to PM2.5 exposure per country. OTHER sources include all emissions except those from residential and road transport (e.g. from the energy sector, industry, agriculture, aviation, shipping). The bullets indicate the contribution (in % of the total) of the anthropogenic carbonaceous aerosols per sector (brown or green) to the total excess mortality when assuming equal (small bullets) and two-fold (larger bullets) toxicity of anthropogenic carbonaceous aerosols and other PM2.5.

We estimate that with a complete removal of residential combustion emissions (RES100 experiment), the excess mortality would be reduced to 208,000 (CI 159,000–217,000) deaths per year for the European domain. Thus, we find that on average 72,000 (CI 59,000–121,000) excess deaths could be avoided if residential emissions are totally removed (BASE-RES100). From this number, 29,000 (CI 19,000–40,000) deaths are attributed to anthropogenic carbonaceous aerosols (assuming equal toxicity). When anthropogenic carbonaceous aerosols are considered to be twice as toxic, then the benefit in terms of avoidable excess mortality almost doubles to 58,000 (CI 39,000–79,000) avoidable deaths per year, which is almost 80% of the sectoral total due to PM2.5. Although carbonaceous aerosols constitute about 20% of the PM2.5 mass, much less than the inorganic mass fraction, due to their higher toxicity, they may account for the largest proportion of excess mortality. These results highlight the major health benefit that can be achieved by removing or mitigating carbon-rich emission sources, potentially much more than assumed thus far, since differential toxicity of carbonaceous aerosols is typically not accounted for in health impact studies. This is primarily due to inconclusive epidemiological evidence on the relationship between those particles and health effects, mostly due to the lack of aerosol speciation data from air quality monitoring networks. Furthermore, the contribution of residential combustion to total excess mortality varies between countries and can range between about 9% and 39% of the total (Fig. 2). Nevertheless, the total removal of residential combustion emissions is a scenario that is not realistic in the near future. Moreover, it is very difficult to monitor and limit those emissions, especially in rural regions where the activity in individual households is not well recorded^24^.

**Figure 3:**
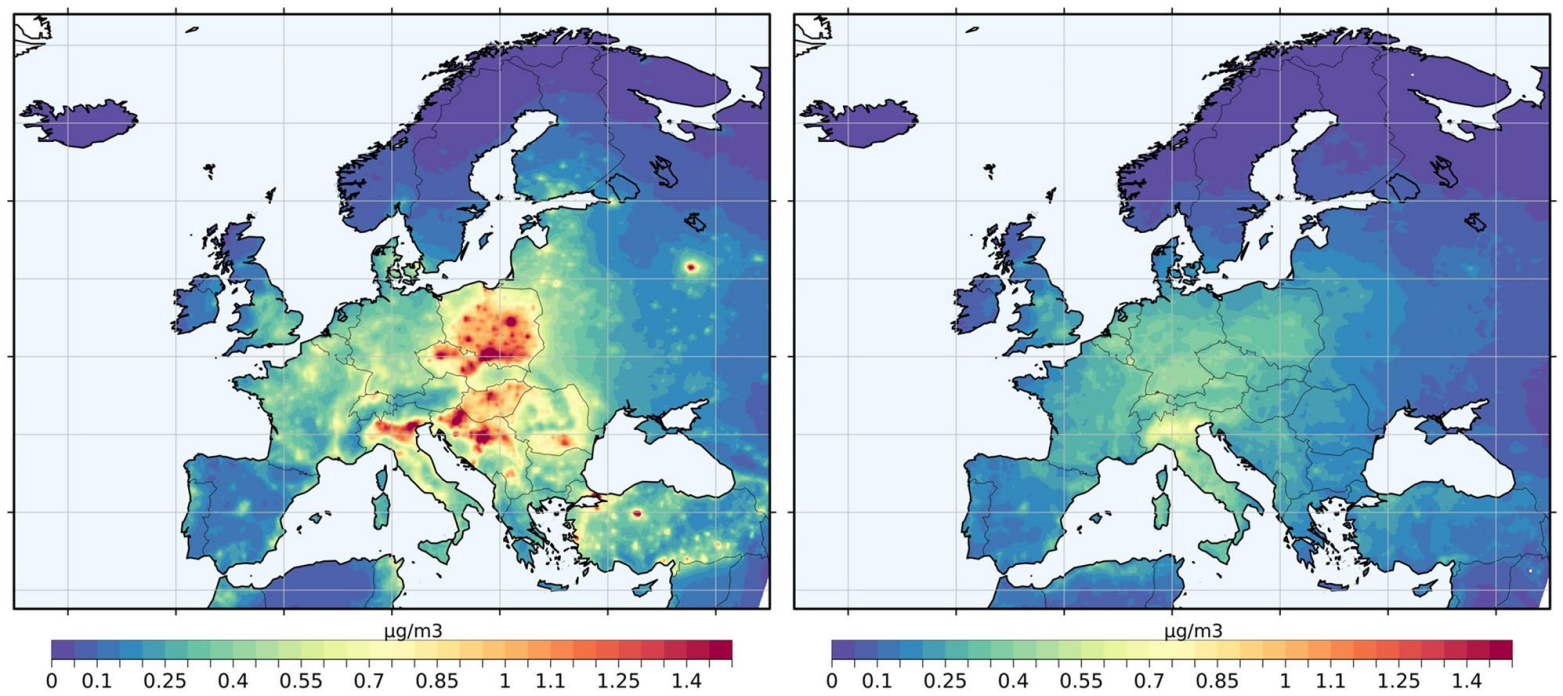
Differences in annual mean PM2.5 concentrations between the baseline and the 20% emission reduction scenarios, for residential combustion (BASE-RES20—left panel) and transport (BASE-TRA20—right panel). This figure was produced with R software, version 4.3.2 (https://cran.r-project.org/bin/windows/base/).

**Figure 4:**
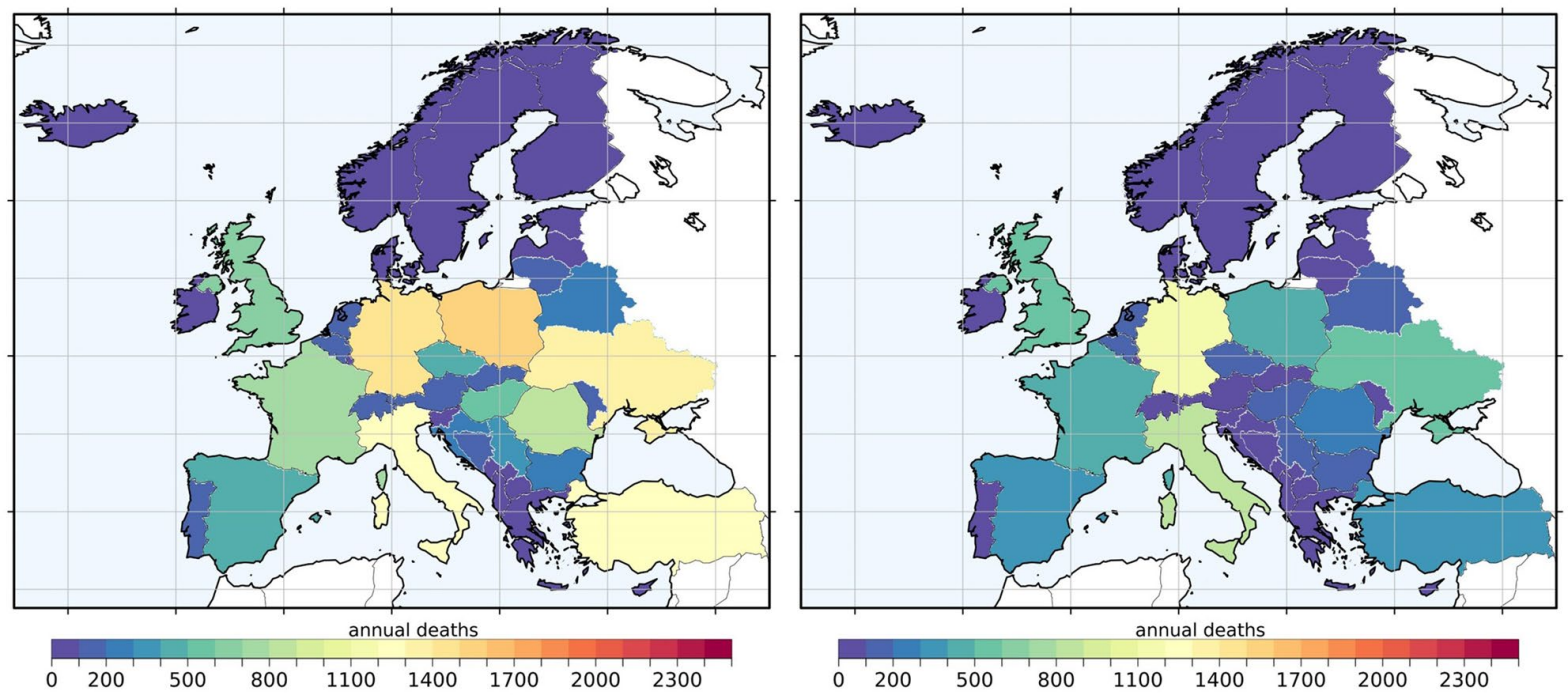
Avoidable excess mortality in each country based on the 20% emission reduction scenarios RES20 (left) and TRA20 (right). This figure was produced with R software, version 4.3.2 (https://cran.r-project.org/bin/windows/base/).

Under the RES20 scenario (20% reduction in residential combustion emissions), we find an annual reduction of up to 1.35 μg/m^3^ in PM2.5 concentration (Fig. 3, left panel). However, the population-weighted exposure reduction ranges between 0.2 and 0.9 μg/m^3^ for most countries and reaches a maximum of 1.2 μg/m^3^ in Easter European countries like Croatia and Poland. With such ambient concentration reductions, the adult excess mortality can be reduced to 268,000 (CI 198,000–299,000) deaths per year for the region. This is a ~ 5% reduction of annual excess mortality compared to the baseline scenario, whereas RES100 results in ~ 26% fewer deaths compared to the baseline scenario. Our results indicate that Central and Eastern Europe would enjoy significant benefits with regards to ambient PM2.5 concentration reductions in the RES20 scenario. The hot spots of PM2.5 air pollution from residential combustion emissions are found in Eastern European countries (e.g. Estonia, Poland, and Serbia), which also achieve the highest reductions in PM2.5 annual concentrations under the RES20 scenario (Fig. 3). Northern Italy may achieve markedly high annual mean PM2.5 reductions as well. However, no country falls below the TMREL threshold of 4.15 μg/m^3^ with such emission reductions, except for Iceland and Norway, where exposure levels are already low. Finland and Sweden are close to the TMREL value (with ~ 4.4 μg/m^3^) and only fall below the TMREL under the RES100 scenario (reduction to ~ 3.9 μg/m^3^). Anthropogenic carbonaceous aerosols follow the same concentration reduction patterns over the model domain for the RES20 scenario. These concentration reductions result in a similar spatial distribution of avoidable excess deaths over the region (Fig. 4).

#### Road transport

We estimate that on average 35,000 (CI 23,000–47,000) adult excess deaths per year could be avoided if road transport emissions were totally removed from the European atmosphere (TRA100), of which 6000 (CI 4000–9000) are due to anthropogenic carbonaceous aerosols (assuming equal toxicity). When we assume anthropogenic carbonaceous aerosols to be twice as toxic, then their contribution increases to 13,000 (CI 8000–17,000) annual excess deaths from the road transport sector, which constitutes 37% of the sectoral total and 4.6% of the overall total.

As mentioned above, TRA100 is not a realistic scenario for the near future and is rather a weighted estimate of the contribution of the road transport sector to excess mortality. Assuming that road transport emissions are reduced by 20% (TRA20), we estimate that a reduction in annual mean PM2.5 concentration of up to 0.83 μg/m^3^ can be achieved (Fig. 3, right panel). These ambient PM2.5 concentration reductions are lower than those from the RES20 scenario and are more equally distributed in central Europe. Northern Italy would achieve the highest benefit in annual PM2.5 concentration reductions in the TRA20 scenario. Anthropogenic carbonaceous aerosols follow the same pattern in concentration reductions. With such emission reductions (TRA20), we estimate an annual excess mortality of 274,000 (CI 203,000–308,000). This is a ~ 3% reduction compared to the baseline scenario and equals approximately 7000 (4000–9000) avoidable excess deaths per year for the whole region. At the country level, this reduction ranges between 1 and 4%, with Spain having the highest reduction in excess mortality under the TRA20 scenario. This equals to 4% or 369 (227–525) of the excess deaths per year (Table 3). Taking into account the population numbers in each country, Serbia, Ukraine, and Hungary remain at the top of the list with the highest population-normalized excess mortalities due to PM2.5 exposure under the TRA20 scenario (Table S3). The respective population-weighted results under the RES20 scenario in each country are similar with the TRA20 scenario. Overall, the per-country reductions in excess mortality compared to the BASE scenario are relatively small and range between 2 and 8%. However, we cannot ignore the significant health impacts of this specific source category. Road transport emissions, specifically diesel and gasoline exhaust emissions, which include carbon-rich and relatively hazardous fine particles^11^, are classified as carcinogenic (Group1) and possibly carcinogenic, respectively (Group2B) in humans (IARC, 2016). Here, we estimate that one fifth of the road transport-related excess mortality is due to lung cancer. This equals 7000 (CI 5000–10,000) excess deaths per year, of which up to 6000 (CI 4000–8000) can be attributed to the anthropogenic carbonaceous fraction (assuming two-fold higher toxicity).

**Table 3:** Per-country avoidable total excess mortality (sum of the six causes of death in adults) that can be achieved from each emission reduction scenario.

	RES100	RES20	TRA100	TRA20
Albania	225 (135–336)	42 (26–62)	140 (85–208)	27 (16–39)
Austria	710 (469–985)	133 (90–181)	532 (352–740)	99 (66–135)
Belgium	743 (498–1011)	141 (96–189)	671 (451–912)	121 (83–162)
Bosnia and Herzegovina	927 (610–1229)	163 (112–208)	197 (134–255)	37 (25–47)
Bulgaria	1456 (962–1990)	274 (186–366)	585 (393–787)	112 (76–150)
Belarus	1422 (876–2096)	268 (168–386)	764 (475–1114)	146 (91–210)
Croatia	1176 (825–1531)	208 (154–259)	266 (193–336)	50 (36–62)
Cyprus	37 (22–53)	7 (4–10)	26 (16–37)	5 (3–7)
Czech Republic	2401 (1705–3072)	411 (305–506)	727 (530–910)	134 (99–165)
Denmark	394 (245–574)	75 (47–107)	186 (117–268)	34 (21–48)
Estonia	115 (66–208)	22 (13–38)	32 (18–57)	6 (3–10)
Finland	245 (154–283)	48 (30–58)	69 (43–80)	13 (8–15)
France	3828 (2375–5539)	724 (458–1028)	2378 (1486–3419)	438 (277–623)
Germany	7683 (5013–10,659)	1443 (962–1976)	6096 (3992–8440)	1111 (741–1522)
Greece	724 (421–878)	138 (67–158)	393 (204–422)	76 (37–87)
Hungary	2832 (1949–3758)	511 (369–643)	647 (465–822)	122 (89–153)
Iceland	0	0	0	0
Ireland	120 (69–160)	23 (13–32)	69 (40–91)	13 (7–17)
Italy	7121 (4887–9403)	1296 (917–1678)	4311 (2980–5673)	828 (582–1077)
Latvia	367 (229–545)	67 (43–97)	102 (64–150)	19 (12–28)
Liechtenstein	5 (4–7)	1 (0.7–1.2)	1 (0.9–1.5)	0.2 (0.17–0.3)
Lithuania	556 (355–792)	104 (68–144)	224 (145–315)	42 (27–58)
Luxembourg	26 (17–36)	5 (3–7)	29 (19–41)	5 (4–7)
Malta	10 (6–14)	2 (1–2)	13 (8–18)	3 (1–4)
Moldova	612 (383–911)	115 (74–167)	181 (115–264)	35 (22–50)
Montenegro	67 (42–95)	13 (8–18)	36 (23–51)	7 (4–9)
Netherlands	738 (476–1028)	141 (92–194)	814 (525–1136)	146 (95–201)
Norway	108 (70–122)	21 (13–26)	54 (35–61)	11 (6–12)
Poland	9012 (6185–11,904)	1583 (1141–2004)	2233 (1581–2870)	419 (300–531)
Portugal	619 (374–901)	118 (72–172)	429 (260–620)	80 (49–115)
Romania	4488 (3026–6067)	832 (581–1082)	1100 (760–1446)	210 (147–274)
Serbia	1873 (1361–2349)	344 (258–419)	537 (397–662)	101 (75–123)
Slovakia	691 (455–967)	129 (88–177)	303 (203–418)	56 (38–77)
Slovenia	262 (176–364)	48 (33–64)	110 (76–148)	20 (14–27)
Spain	2284 (1386–3222)	436 (268–623)	1978 (1203–2748 (	369 (227–525)
Sweden	295 (180–406)	57 (35–79)	214 (131–297)	40 (24–55)
Switzerland	578 (374–824)	107 (71–148)	384 (250–544)	72 (48–100)
Turkey	7674 (5337–10,012)	1296 (946–1639)	6548 (4422–7670)	323 (225–423)
Ukraine	6881 (4330–9923)	1311 (839–1862)	3095 (1965–4425)	589 (377–837)
North Macedonia	286 (188–390)	53 (36–70)	110 (73–148)	21 (14–27)
United Kingdom	3400 (2227–4704)	654 (434–895)	2826 (1854–3909)	517 (342–708)

## Discussion

Our results support a recent study focusing on Europe, in which residential combustion sources have been found to be the main contributors to PM2.5 pollution and excess mortality, especially over Italy and Eastern Europe^42^. Similar results were reported from a global study that reported that carbonaceous aerosols constitute 20% of PM2.5 mass^6^. However, the relationship between PM2.5 exposure and risk of death is not linear and differs between disease categories. For example, the change in risk of death for type II diabetes (according to MR-BRT) per unit change in PM2.5 concentration is highest at relatively low PM2.5 concentrations (e.g., below 20 μg/m^3^), which means that when PM2.5 levels decline, the benefits from emission reduction measures can proportionally increase. For cardiovascular diseases, the risk of death increases significantly both at low and high PM2.5 concentrations, which also makes it the most significant cause of death from PM2.5 air pollution^1,25^. However, the relationship differs depending on the risk model used^7^. Thus, for sensitivity analysis, we also estimate the excess mortality with the GEMM risk model, which results in two-fold higher mortality estimates compared to MR-BRT (Supplementary material, Table S1). Furthermore, in Chowdhury et al.^6^, the authors used a general circulation model (at roughly 100 × 100 km resolution) to simulate global PM2.5 for the year 2015 and estimated the contribution of several emission sectors to excess mortality using toxicity assumptions. They estimated that in the EU-27, 19% and 10% of the total excess mortality can be attributed to domestic energy use-related emissions and transportation, respectively (assuming equal toxicity). Our results, based on higher-resolution regional simulations, underscore that domestic fuel combustion and the road transport sector contribute 26% and 13% of the total excess mortality, respectively, in the wider European region (assuming equal toxicity).

Furthermore, we assessed whether the PM2.5 concentrations respond linearly in the emission reduction scenarios (Supplementary Information). From this evaluation, we find that the PM2.5 levels attributable to each sector are almost equal to five times the PM2.5 under the conditions in which 20% of the respective sectoral emissions remain (RES = 5 × 20%RES and TRA = 5 × 20%TRA) (Figure S1). This underscores that PM2.5 responds almost linearly to emission reductions, indicative of little influence from other components. Our results confirm that applying emission reduction scenarios is an appropriate method to quantify sectoral contributions, which is significant for future studies that intend to use this approach.

## Uncertainties and limitations

The anthropogenic emissions included in our model are representative of the year 2015; therefore, the estimated PM2.5 concentrations and excess mortality reductions from our emissions scenarios are comparable to the activity data reported for that year. Additional simulations with updated emissions would be useful for considering the emission changes that have occurred since 2015 (e.g., due to mitigation policies, weather patterns, health- and population-associated changes). Furthermore, considering uncertainties in emission inventories, reduction scenarios might not fully reflect the actual avoidable mortality due to PM2.5 air pollution. For example, condensable organic particles, which are known to contribute significantly to secondary organic aerosol formation and total PM2.5, are not reported consistently by countries, and thus, are not well represented in emission inventories^24^. A previous study estimated that modeling uncertainties, including those arising from incomplete emission data, result in 15% deviations in model calculated excess mortality in Europe^33^. Furthermore, there are other sources that emit carbon-rich and relatively toxic particles, being particularly harmful to human health, such as the use of coal in industry and power plants^11,43^. Due to limitations in computational resources, we restricted our model simulations to only two PM2.5 emission sectors, residential combustion and road transport, which are key PM2.5 sources in Europe, known to be responsible for leading health impacts. Furthermore, since the true magnitude of the relative toxicity of carbonaceous aerosols is still not robustly determined in epidemiological studies, additional work on speciated PM2.5 toxicity would be beneficial for policy making.

## Conclusions

In this study, by utilizing a regional air quality model and based on four emission scenarios, we estimated the contributions of the residential combustion and road transport sectors to excess mortality due to PM2.5 exposure in Europe. For the year 2015, a representative and well-studied period, we estimate that 72,000 (CI 48,000–99,000) and 35,000 (CI 23,000–47,000) excess deaths per year could be avoided if residential and road transport emissions, respectively, would be phased out. Depending on the relative toxicity assumption, 29,000–58,000 and 6000–13,000 annual excess deaths are attributed to anthropogenic carbonaceous aerosols from residential combustion sources and road transport, respectively. Based on a realistic scenario for the near future, under which a 20% reduction in these emissions is applied (RES20, TRA20), about 13,000 (CI 9000–18,000) and 7000 (CI 4000–9000) excess deaths per year could be avoided, respectively.

Further, we evaluated our emission reduction method for its suitability to determine the relative contributions of different sectors to PM2.5 concentrations. We find that PM2.5 resulting from emissions by these sectors responds nearly linearly in mitigation scenarios, which suggests that the sequential emission reduction method can be considered appropriate. Although we assessed this outcome for the two aforementioned sectors, our results are important for future studies that aim to use the same approach for a sectoral air pollution assessment. Finally, we identified country-specific impacts from the sector contributions to excess mortality, which underscore the importance of nationally oriented emission reduction strategies. Nevertheless, coordinated EU policies are needed to address the long-range transport of air pollution.

### Supplementary Information

Supplementary Information.

## Data Availability

Data will be available from the corresponding author on a reasonable request.
