# Supplementary material for: Health effects of carbonaceous PM2.5 compounds from residential fuel combustion and road transport in Europe
Source: Sci Rep. 2024 Jan 17;14:1530. doi: 10.1038/s41598-024-51916-9 (PMC10794246; doi:10.1038/s41598-024-51916-9)
Supplement: Supplementary file 1 — Supplementary Information. [file 41598_2024_51916_MOESM1_ESM.pdf]

Health effects of carbonaceous PM2.5 compounds from residential fuel combustion and road transport in Europe

Niki Paisi[a*], Jonilda Kushta[a], Andrea Pozzer[a,b], Angelos Violaris[a], Jos Lelieveld[a,b]

[a]Climate and Atmosphere Research Center (CARE-C), The Cyprus Institute, 2121 Nicosia, Cyprus

[b]Department of Atmospheric Chemistry, Max Planck Institute for Chemistry, 55128 Mainz, Germany

*Corresponding author: Niki Paisi (n.paisi@cyi.ac.cy)

Supplementary Material

1. The Global Exposure Mortality Model (GEMM)

1.1 Methodology

Gridded adult (>25 years) and children's (<5 years) population data for the year 2015 from SEDAC, GPW-v4 (https://sedac.ciesin.columbia.edu), were combined with country-level age information from the Global Burden of Disease (GBD, 2017) to obtain age specific gridded population estimates for the following age classes: above 25 years and at 5 year intervals: 25-29, 30-34, 35-39, 40-44, 45-49, 50-54, 55-59, 60-64, 65-69, 70-74, 75-79 and 80 plus. BMRs were obtained for each of the diseases (including non-communicable diseases (NCD), ischemic heart disease (IHD), stroke, lung cancer (LC), lower respiratory infections (LRI), chronic obstructive pulmonary disease (COPD). Age and disease depended Hazard Ratios (HR) groups in adults were derived by the Global Exposure Mortality Model (GEMM) (eq.1) (Burnett et al., 2018)

$$HR\,(z) = \ exp\left(\theta \times log\left(\left(\frac{z}{a}\right) + 1\right) \Big/ \left(1 + exp\left(-\frac{z-\mu}{v}\right)\right)\right),$$

$$\mathrm{z = max(0, PM2.5 - 2.4\mu g/m3)} \qquad \text{(eq. 1)}$$

where, z is the annual mean $PM_{2.5}$ concentration for the year 2015 and 2.4μg/m^3 is the counterfactual concentration below which no health risk is assumed (Burnett et al., 2018). θ, a, μ, ν are the parameters of the fit for the model obtained from Burnet et al. (2018) supplementary information. The HR were used to calculate the attributable fraction (in equations 2 and 3), which equals to HR-1/HR. We performed all the calculations at the population's grid level (5×5km) and then we aggregated to the country and the European level.

$$M_{PM2.5} = \ BMR \ \times \ POP \ \times \ \mathrm{HR - 1/HR} \qquad \text{(eq. 2)}$$

$$M_{aCA} = M_{PM2,5} \times \left(\frac{CA}{PM2.5}\right) \qquad \text{(eq.3)}$$

1.2. Excess mortality results

Table S1: Excess mortality per country due to PM2.5 and anthropogenic carbonaceous aerosols (aCA) assuming equal and double toxicity using the GEMM model rounded to the nearest thousand (baseline scenario).

	**PM2.5**			**aCA (Equal toxicity)**			**aCA (two-fold toxicity)**		
Country	Average	Lower limit	Upper limit	Average	Lower Limit	Upper Limit	Average	Lower Limit	Upper Limit
Albania	2300	1600	3100	300	200	400	600	400	800
Austria	8400	6900	9900	1300	1100	1600	2700	2200	3100
Belgium	11600	9500	13700	2000	1600	2300	3900	3200	4600
Bosnia & Herzegovina	4700	3900	5500	900	700	1000	1800	1400	2100
Bulgaria	11700	9600	13800	1800	1500	2200	3700	3000	4300
Belarus	10300	8500	12200	1600	1300	1900	3100	2600	3700
Croatia	6400	5200	7500	1300	1100	1500	2600	2100	3000
Cyprus	700	600	900	100	100	100	100	100	200
Czech Rep.	14200	11600	16600	3100	2500	3600	6100	5100	7200
Denmark	4400	3600	5300	600	500	700	1200	1000	1500
Estonia	1100	900	1400	200	100	200	400	300	400
Finland	3600	2900	4200	600	500	700	1100	900	1300
France	49400	40600	58200	8100	6700	9500	16200	13300	19100
Germany	99900	82500	117200	15300	12700	18000	30700	25300	36000
Greece	12100	9900	14300	1600	1300	1900	3200	2600	3800
Hungary	16200	13300	18900	3500	2900	4100	7000	5700	8100
Iceland	35	28	43	1	1	2	3	2	3
Ireland	1900	1500	2200	200	200	200	400	300	500
Italy	71800	59400	84100	12300	10100	14400	24500	20300	28700
Latvia	2500	2000	3000	400	300	500	800	700	1000
Liechtenstein	30	25	36	6	5	7	13	11	15
Lithuania	3900	3200	4600	700	600	800	1300	1100	1600
Luxembourg	400	300	500	100	100	100	100	100	200
Malta	300	200	300	27	22	33	54	44	100
Moldova	3900	3200	4600	700	600	900	1400	1200	1700

Montenegro	600	500	800	100	100	100	200	100	200
Netherlands	14000	11500	16500	2000	1600	2400	4000	3300	4700
Norway	2100	1700	2500	200	200	200	400	300	500
Poland	49500	40800	58000	11000	9000	12800	21900	18100	25700
Portugal	8300	6800	9800	1100	900	1300	2200	1800	2600
Romania	29000	23900	34000	5500	4500	6400	11000	9000	12900
Serbia	13900	11500	16300	2500	2100	3000	5000	4200	5900
Slovakia	6200	5100	7300	1000	800	1200	2000	1700	2400
Slovenia	2200	1800	2700	400	300	500	800	600	900
Spain	32100	26400	37800	4900	4100	5800	9900	8100	11600
Sweden	6200	5100	7400	800	600	900	1500	1300	1800
Switzerland	6100	5000	7200	900	800	1100	1900	1500	2200
Turkey	48500	38400	59200	8400	6700	10300	16900	13400	20600
Ukraine	65900	54300	77400	10400	8500	12200	20700	17100	24300
North Macedonia	2000	1700	2400	300	200	400	600	500	700
United Kingdom	52000	43100	60900	7800	6500	9200	15700	13000	18400

2. The Meta-regression-Bayesian, Regularized, Trimmed (MR-BRT) exposure response function

2.1 Excess mortality results

Table S2: The contribution of anthropogenic carbonaceous aerosols (aCA) to total excess mortality (in percentage) per country (baseline scenario and based on MR-BRT).

Country	% contribution of aCA to PM2.5 Equal toxicity	% contribution of aCA to PM2.5 Double toxicity
Albania	13.1	26.2
Austria	16.5	33.1
Belgium	17.2	34.4
Bosnia & Herzegovina	19.1	38.2
Bulgaria	16.2	32.5
Belarus	15.7	31.5

Croatia	20.7	41.4
Cyprus	9.7	19.4
Czech Republic	22.2	44.4
Denmark	14.2	28.5
Estonia	16.5	33.1
Finland	17.3	34.7
France	16.9	33.9
Germany	15.8	31.6
Greece	13.7	27.5
Hungary	22.0	44.0
Iceland	5.7	11.3
Ireland	11.4	22.7
Italy	17.6	35.3
Latvia	17.5	35.0
Liechtenstein	21.5	43.0
Lithuania	17.7	35.5
Luxembourg	17.0	33.9
Malta	10.5	21.0
Moldova	19.1	38.1
Montenegro	13.0	26.0
Netherlands	14.5	29.1
Norway	10.9	21.8
Poland	22.7	45.4
Portugal	13.6	27.3
Romania	19.5	39.0
Serbia	18.5	37.0
Slovakia	16.9	33.7
Slovenia	18.2	36.5
Spain	16.0	32.0
Sweden	13.2	26.3
Switzerland	16.0	32.0
Turkey	18.0	36.0
Ukraine	16.1	32.3
North Macedonia	15.3	30.6
United Kingdom	15.6	31.2

Table S3: Population normalized excess mortality due to PM2.5 under the TRA20 scenario (based on MR-BRT).

**Country**	**Excess deaths per 100,000 population**
Albania	31

Austria	23
Belgium	19
Bosnia & Herzegovina	43
Bulgaria	54
Belarus	45
Croatia	48
Cyprus	15
Czech Republic	48
Denmark	10
Estonia	22
Finland	11
France	10
Germany	25
Greece	26
Hungary	56
Iceland	0
Ireland	5
Italy	21
Latvia	40
Liechtenstein	28
Lithuania	52
Luxembourg	15
Malta	21
Moldova	42
Montenegro	30
Netherlands	11
Norway	4
Poland	39
Portugal	8
Romania	44
Serbia	66
Slovakia	43
Slovenia	22
Spain	8
Sweden	10
Switzerland	15
Turkey	22
Ukraine	64
North Macedonia	27
United Kingdom	11

Table S4: Population weighted mean concentrations per country and scenario (µg/m^3).

Country	**BASE**	**RES20**	**TRA20**	**RES100**	**TRA100**

	PM25	aCA	PM25	aCA	PM25	aCA	PM25	aCA	PM25	aCA
Albania	14.5	1.9	14.0	1.7	11.7	2.1	12.1	1.0	13.0	1.7
Austria	14.2	2.3	13.6	2.1	13.5	2.1	7.7	0.7	9.0	1.3
Belarus	10.1	1.6	9.7	1.4	13.2	1.7	13.3	1.7	13.5	2.1
Belgium	15.6	2.7	15.1	2.5	10.9	1.9	12.7	1.2	17.1	3.3
Bosnia and Herzegovina	18.4	3.5	17.3	3.1	15.2	2.6	10.9	1.2	12.5	2.0
Bulgaria	13.6	2.2	13.0	2.0	13.9	1.8	8.1	0.8	9.0	1.4
Croatia	17.9	3.7	16.7	3.2	14.2	1.8	11.7	1.2	16.4	3.5
Cyprus	13.8	1.3	13.5	1.3	9.1	1.4	12.7	1.0	13.0	1.2
Czech Republic	19.3	4.3	18.1	3.8	13.8	2.3	13.2	1.7	17.3	3.9
Denmark	10.8	1.5	10.4	1.4	10.8	2.1	8.8	0.7	9.8	1.4
Estonia	9.6	1.6	9.2	1.4	15.3	2.5	7.4	0.7	9.0	1.5
Finland	8.2	1.3	7.9	1.2	15.2	2.6	6.8	0.8	7.8	1.3
France	12.1	2.0	11.6	1.8	16.1	2.8	9.7	1.1	10.6	1.7
Germany	13.9	2.2	13.4	2.0	17.7	3.8	11.5	1.2	12.0	1.8
Greece	14.1	1.9	13.8	1.7	17.2	3.9	12.5	1.2	13.2	1.7
Hungary	17.1	3.8	16.0	3.3	18.2	3.5	11.5	1.5	15.7	3.5
Iceland	3.2	0.1	3.2	0.1	10.4	1.6	3.2	0.1	3.2	0.1
Ireland	7.1	0.8	6.9	0.7	7.9	1.0	6.2	0.4	6.6	0.7
Italy	16.5	2.9	15.8	2.6	18.9	4.2	12.7	1.4	14.2	2.6
Latvia	11.1	1.9	10.5	1.7	3.2	0.1	8.4	0.8	10.3	1.8
Liechtenstein	18.0	3.9	16.8	3.4	9.5	1.4	12.2	1.5	16.5	3.6
Lithuania	11.9	2.1	11.4	1.9	11.8	2.0	9.1	1.0	10.8	1.9
Luxembourg	15.8	2.7	15.4	2.5	7.0	0.7	13.4	1.7	13.1	1.9
Malta	12.5	1.3	12.3	1.2	13.7	2.2	11.5	1.0	11.3	1.1
Moldova	11.0	2.1	10.5	1.9	16.8	3.7	8.3	1.0	10.2	1.9
Montenegro	13.4	1.7	13.0	1.6	12.2	1.3	11.3	0.9	12.2	1.6
Netherlands	13.0	1.9	12.6	1.7	14.4	2.8	11.4	1.2	11.2	1.5
North Macedonia	14.9	2.3	14.3	2.0	8.1	1.3	5.5	0.4	5.9	0.6
Norway	6.2	0.6	6.1	0.6	14.7	2.2	11.4	1.4	15.9	3.7
Poland	17.5	4.0	16.3	3.5	6.6	0.6	8.2	0.8	8.6	1.1
Portugal	9.5	1.3	9.2	1.2	17.0	3.1	10.7	1.2	13.6	2.7
Romania	14.6	2.8	13.9	2.5	13.6	1.3	6.6	0.8	7.9	1.4
Serbia	20.8	3.8	19.8	3.4	17.6	3.7	12.2	1.3	14.0	2.4
Slovakia	15.5	2.6	14.8	2.4	10.6	1.5	12.4	1.1	15.2	2.8
Slovenia	17.4	3.2	16.4	2.8	9.9	1.5	7.9	0.9	8.1	1.2
Spain	9.3	1.5	9.1	1.4	20.5	3.8	7.2	0.7	7.5	0.9
Sweden	8.0	1.0	7.9	1.0	18.3	3.3	10.9	1.0	11.9	1.9
Switzerland	14.1	2.2	13.5	2.0	9.5	1.5	13.2	1.6	17.4	3.2
Turkey	18.6	3.4	17.5	3.0	13.3	2.2	8.9	1.1	9.7	1.6

Ukraine	10.4	1.7	10.1	1.6	8.2	1.5	12.0	1.1	13.7	2.1
United Kingdom	10.6	1.6	10.3	1.5	9.3	1.3	9.0	1.0	9.3	1.3

3. Emission reduction scenarios and the contribution of residential combustion (RES) and road-transport (TRA) to PM2.5 concentration.

We estimate the contribution of each sector (RES or TRA) to PM2.5 concentration by removing the PM2.5 under the 100% emission reduction scenario (e.g. RES100 or TRA100) from the baseline scenario (e.g. BASE-RES100 or BASE-TRA100). If the exposure-response relationship is linear, the PM2.5 concentration that is due to each sector (RES or TRA) should be equal to five times the PM2.5 concentration under the scenario where only 20% of the sectoral emissions remain (BASE-RES20 or BASE-TRA20). To assess if this assumption is valid, we divided the PM2.5 concentration from each sector (RES or TRA) by the 5×(BASE-RES20), and assuming linearity the quotient should be equal to one. We performed these calculations for each grid box and plotted the quotients. We find that the numbers for most of the region are very close to one, indicating that the divisor and dividend are almost equal. This indicates that the system is almost linear for residential and road transport emissions and that the health impacts from these sources are not strongly affected by other atmospheric components.

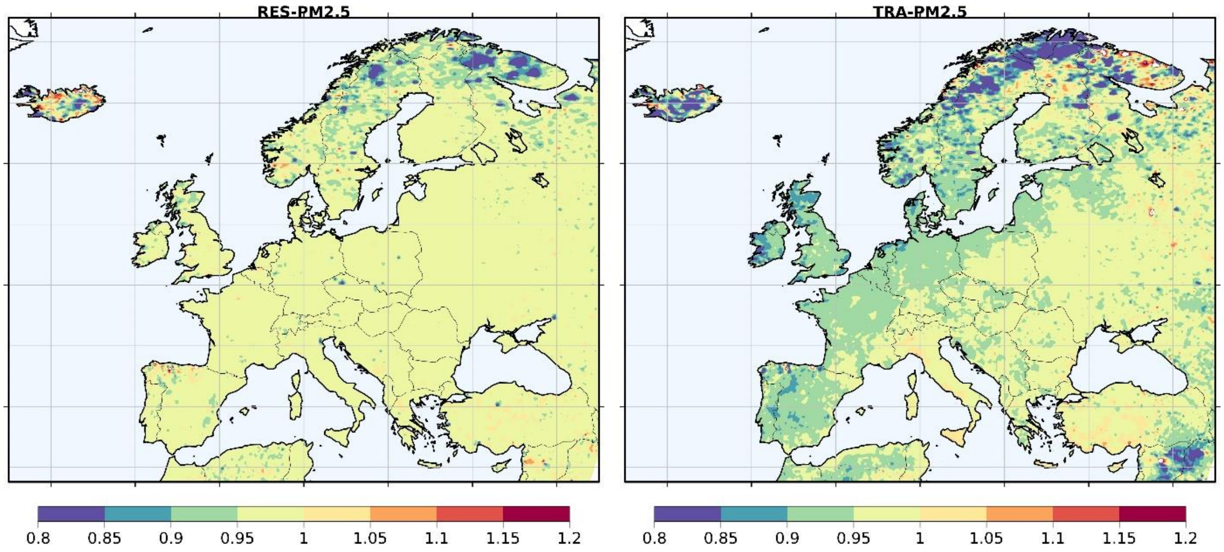

Figure S1: PM2.5 concentration from each sector (RES, left panel and TRA, right panel) divided with the respective PM2.5 concentration from BASE-RES20 simulation multiplied by 5. The closer the values to one indicates that the two resulting concentrations are nearly equal (RES=5×20%RES and TRA=5×20%TRA). This figure was generated with R software, version 4.3.2 (https://cran.r-project.org/bin/windows/base/).

4. WRF-Chem model evaluation.

The simulated annual mean PM2.5 concentrations were compared against available observations. The observations were obtained from the EBAS-Nilu (http://ebas.nilu.no/) and AirBase (https://www.eea.europa.eu/) databases. In total, 996 stations that cover a large part of Europe were used for the comparison. The comparison was performed for the annual PM2.5 averages, which are relevant to long-term exposure and are used as input to the MR-BRT and the GEMM risk models. In the baseline scenario, we estimate an annual mean bias of -0.7 μg/m^3, which shows that the model tends to underestimate the observed PM2.5 concentrations. This is mostly evident at the higher concentration levels, above 30 μg/m^3, hence the overall influence is small (Figure S2). The model bias is well below the uncertainties associated with the observational dataset, which is 10-15%. Based on the criteria of Boylan and Russell (2006), the model mean fractional bias (MFB) and error (MFE) should be below 30% and 50%, respectively, to be considered as the best that can be expected in terms of model accuracy (Boylan & Russell, 2006). In this work, we estimate a MFB and MFE for PM2.5 equal to -1% and 21%, respectively.

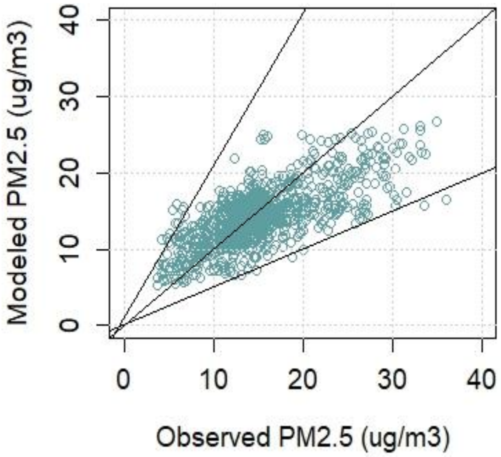

Figure S2: Annual mean modeled PM2.5 concentrations in comparison with observations.

Table S5: Model performance metrics for PM2.5 in the baseline simulation.

PM2.5	Mean Bias	Mean Error	Mean Fractional Bias	Mean Fractional Error
	-0.7 μg/m^3	3 %	-1 %	21 %

The model performance metrics were calculated as follows:

Mean Bias = $\frac{1}{n}\sum_{1}^{n}(M - O)$

Mean Error = $\frac{1}{n}\sum_{1}^{n}|M-O|$

Fractional Bias = $\frac{1}{n}\left(\frac{\sum_{1}^{n}(M-O)}{\sum_{1}^{n}\left(\frac{(M+O)}{2}\right)}\right)$

Fractional Error = $\frac{1}{n}\left(\frac{\sum_{1}^{n}|M-O|}{\sum_{1}^{n}\left(\frac{(M+O)}{2}\right)}\right)$

M: modeled concentration, O: observed concentration, n: number of observations.

References

Boylan, J. W., & Russell, A. G. (2006). PM and light extinction model performance metrics, goals, and criteria for three-dimensional air quality models. *Atmospheric Environment*, *40*(26), 4946–4959. https://doi.org/10.1016/J.ATMOSENV.2005.09.087

Burnett R., Chen H., Szyszkowicz M., Fann N., Hubbell B., Pope A., Apte J., Brauer M., Cohen A., Weichenthal S., Coggins J., Di Q., Brunekreef B., Frostad J, Lim S., Walker K., Spadaro J. Thurston G. D., Hayes R. B., Lim C., Turner M., Jerret M., Krewski, S. J. V. (2018). Global estimates of mortality associated with longterm exposure to outdoor fine particulate matter. *Proceedings of the National Academy of Sciences of the United States of America*, *115*(38), 9592–9597. https://doi.org/10.1073/pnas.1803222115

Global Burden of Disease Study. (2017). In *The Lancet*. http://ghdx.healthdata.org/record/ihme-data/gbd-2017-population-estimates-1950-2017
